# Feature-based molecular networking for identification of organic micropollutants including metabolites by non-target analysis applied to riverbank filtration

**DOI:** 10.1007/s00216-021-03500-7

**Published:** 2021-07-20

**Authors:** Daniela Oberleitner, Robin Schmid, Wolfgang Schulz, Axel Bergmann, Christine Achten

**Affiliations:** 1 Institute of Geology and Palaeontology – Applied Geology, University of Münster, Corrensstraße 24, 48149 Münster, Germany; 2 Institute of Inorganic and Analytical Chemistry, University of Münster, Corrensstraße 28/30, 48149 Münster, Germany; 3 Laboratory for Operation Control and Research, Zweckverb and Landeswasserversorgung, Am Spitzigen Berg 1, 89129 Langenau, Germany; 4 Rheinisch-Westfälische Wasserwerksgesellschaft mbH, Am Schloß Broich 1-3, 45479 Mülheim (Ruhr), Germany

**Keywords:** Identification of unknowns, Molecular networking, Transformation products, Tandem mass spectrometry, Environmental analysis

## Abstract

Due to growing concern about organic micropollutants and their transformation products (TP) in surface and drinking water, reliable identification of unknowns is required. Here, we demonstrate how non-target liquid chromatography (LC)-high-resolution tandem mass spectrometry (MS/MS) and the feature-based molecular networking (FBMN) workflow provide insight into water samples from four riverbank filtration sites with different redox conditions. First, FBMN prioritized and connected drinking water relevant and seasonally dependent compounds based on a modification-aware MS/MS cosine similarity. Within the resulting molecular networks, forty-three compounds were annotated. Here, carbamazepine, sartans, and their respective TP were investigated exemplarily. With chromatographic information and spectral similarity, four additional TP (dealkylated valsartan, dealkylated irbesartan, two oxygenated irbesartan isomers) and olmesartan were identified and partly verified with an authentic standard. In this study, sartans and TP were investigated and grouped regarding their removal behavior under different redox conditions and seasons for the first time. Antihypertensives were grouped into compounds being well removed during riverbank filtration, those primarily removed under anoxic conditions, and rather persistent compounds. Observed seasonal variations were mainly limited to varying river water concentrations. FBMN is a powerful tool for identifying previously unknown or unexpected compounds and their TP in water samples by non-target analysis.

**Graphical abstract:**

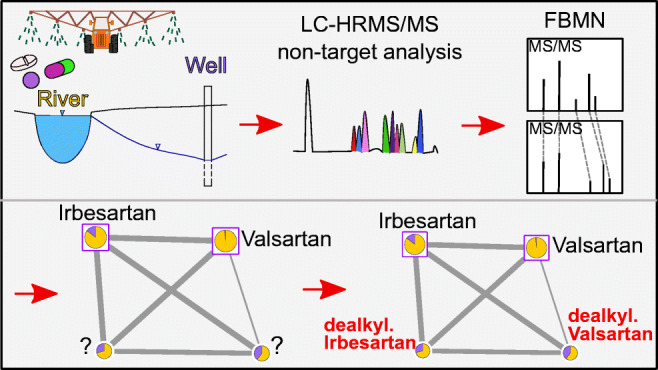

**Supplementary Information:**

The online version contains supplementary material available at 10.1007/s00216-021-03500-7.

## Introduction

Due to rising concern about the increasing number of chemical compounds present in our surface waters, the identification of organic micropollutants (OMP) and their transformation products (TP) is necessary [1]. Non-target screening theoretically allows researchers to detect hundreds of chemical compounds in a single analytical run by chromatography coupled to mass spectrometry (MS). However, identifying unknown OMP in environmental water samples is challenging and requires reliable data evaluation [2]. Initially, non-target screening does not require reference standards as compound annotation usually relies on public spectral reference libraries or in silico prediction. However, to turn an annotation into an identification, orthogonal identifiers (e.g., retention time or fragment spectra) are commonly compared to reference standards [3]. Generally, several approaches for the subsequent steps of non-target screening, namely feature detection (e.g., MZmine, enviMass), statistical analysis/prioritization (e.g., MetaboAnalyst, Matlab), and compound annotation/identification (e.g., GNPS, FOR-IDENT, MassBank) in data evaluation of large MS datasets in this context have been made. Prioritization of features (defined by mass-to-charge ratio (m/z), retention time, and intensity) offers the possibility to focus on a predefined compound group (e.g., formation during a process [4]) and therefore enables a pre-selection of features of interest. A subsequent spectral database search allows the annotation of compounds [2]. Notably, the outcome of this approach is dependent on the availability of reference spectra and therefore falls short for many TP [5]. As a solution to similar challenges, molecular networking (MN) was introduced in 2012 [6] and made publicly available through the Global Natural Products Social Molecular Networking (GNPS) web-platform (http://gnps.ucsd.edu) [7]. The recently published MN protocol provides an introduction to the topic [8]. MN creates networks of nodes, i.e., fragmentation spectra (MS/MS), which are connected based on a pairwise spectral alignment and similarity scoring of all experimental MS/MS spectra in a study, which are often acquired in data-dependent fragmentation [8]. In a second step, nodes are annotated by matching against the public GNPS spectral libraries. This workflow was applied to different non-target MS studies in various fields, including ocean water [9], agricultural [10], forensic [11], and biomedical research [12]. Despite growing MS/MS spectral libraries, annotation rates of only 5% of all experimental MS/MS spectra are common [8]. Nevertheless, many novel compounds were identified with this approach [13–15] and annotations can be propagated throughout a molecular network [16]. The GNPS platform combines multiple tools to analyze and enrich annotations in molecular networks [8, 17, 18]. Compared to classical molecular networking, where all MS/MS spectra are clustered with no regard to retention time, feature-based molecular networking (FBMN) uses only MS/MS spectra that originate from features with a chromatographic peak shape and therefore also considers retention times and peak areas. FBMN enables the identification of isobaric isomers and a more precise statistical evaluation of datasets [19]. Nevertheless, FBMN has so far mainly been used in natural product research and has not been extended towards surface and drinking water TP identification which is shown in this study. In environmental analysis, it has been used to identify TP of anthropogenic source in ocean water [20].

Riverbank filtration (RBF) is a long-used and efficient natural clean-up technique for the effective removal of OMP, bacteria, and other unwanted substances from raw water used for drinking water production [21]. OMP are transported in the groundwater and either adsorbed, transformed, or degraded during RBF and, therefore, specifically either removed or converted to other chemical structures of unknown risk. RBF performance for OMP removal and degradation is dependent on a variety of factors, such as redox conditions, travel time and distance, hydraulic conductivity, or initial OMP concentration [22–25]. Although the behavior during RBF is well studied for many compounds, e.g., commonly used pesticides [26, 27] and pharmaceuticals [28, 29], various TP still lack data on the removal during RBF and suitable removal conditions.

Therefore, we applied FBMN on a set of seasonal RBF samples from four sites at two rivers in Germany to identify unknown compounds and possible TP with non-target liquid chromatography (LC)-MS analysis. After a spectral database search, TP were identified by their spectral similarity to reference standards, data from the literature, and manual spectral evaluation. Furthermore, the identified compounds were statistically analyzed regarding their seasonal occurrence and behavior at the different sites under different redox conditions and travel distances. Therefore, the aims of this study were (1) to implement the application of FBMN on environmental samples and (2) to broaden the knowledge on OMP and TP behavior during RBF.

## Materials and methods

### Sample material and study sites

Four RBF sites in Germany, two at the Ems river and two at the Ruhr river, were investigated (see Supplementary Information (ESM) Figure S1). These sites were previously characterized and chemically analyzed by target analysis [25]. Grab water samples were taken from a transect comprising of river water (R), three groundwater wells (B1-3), and the abstraction well (W) at each RBF site at three different sampling times each: summer 2017 (Su, low discharge), fall 2017 (Fa, medium discharge), and spring 2018 (Sp, low discharge). The Ems river sites (Ea and Eb) consist of anoxic silty sand aquifers with medium conductivity (10^−4^ m/s) and travel distances of 633 and 89 m, respectively. Oxic gravel aquifers characterize the Ruhr river sites (Ra and Rb) with high conductivity (10^−2^ m/s) and travel distances of 72 and 42 m, respectively. The Ems river is predominantly influenced by agricultural activity and wastewater treatment plants (WWTP), whereas the Ruhr river is mainly affected by WWTP, industry, and urban runoff but less agricultural activity. The proportion of treated wastewater (TWW) at mean minimum discharge reaches 50–100% at both rivers, being higher in Ems river [30]. Detailed information on the fraction of treated wastewater in both rivers and effects of discharge can be found in our previous study Oberleitner et al., 2020 [25].

### Analysis of organic micropollutants

The detailed method is described in Oberleitner et al., 2020 [25]. In brief, the samples were acidified to 0.1% formic acid (99.8%, FA from Merck, Germany) and centrifuged at 4000*g* for 10 min, and 490 μL was directly co-injected with 10 μL internal standard (d6-sulfadimethoxine (Dr. Ehrenstorfer), d6-diuron (Campro Scientific), d4-benzotriazole (Chiron AS)) to the HPLC system (Prominence UFLC, Shimadzu, Japan). A binary gradient consisting of acetonitrile (5% water, 0.05% formic acid) and water (0.05% formic acid) was applied on a NucleoShell RP 18plus (Macherey-Nagel, Germany) reversed-phase column and detection was achieved by a Bruker MaXis 3G qTOF tandem mass spectrometer equipped with an electrospray ionization source in positive ionization mode (*m*/*z* 50–1000). By injecting a solution of sodium formate directly into the ESI source before each measurement, mass recalibration was performed. Data-dependent fragmentation was applied, selecting the eight most intensive ions above 400 counts for fragmentation with 35 eV and active exclusion after five spectra for 0.15 min. Mass recalibration and semi-quantitative analysis were performed with DataAnalysis 4.2 (Bruker Daltonics, Germany). Analytical standards of valsartan, valsartan acid, irbesartan, candesartan, olmesartan, telmisartan, 10-hydroxycarbazepine, and 10,11-dihydro-10,11-dihydroxycarbamazepine were purchased from Neochema and Merck and diluted to 1000, 500, and 100 ng/L with 5% acetonitrile (0.1% formic acid) and measured with the same method as the samples.

### Data processing and identification of components

Data files were converted to .mzML file format with CompassXport (Bruker Daltonics, Germany) and processed with MZmine 2.51 [31]. Detailed information on data processing can be derived from ESM Table S1. All data files were grouped by the attributes “Sample Type” (either “River” or “Well”) and “Season” (“Summer,” “Fall,” and “Spring”). Aligned feature lists were filtered to exclude features lacking fragmentation spectra or ^13^C isotope pattern as they would not match the later applied selection criteria due to signal intensity or number of fragments. The resulting lists were exported for FBMN on GNPS. Nevertheless, it cannot be excluded that the lists still contain e.g. adducts or in-source fragments as multiple signals from only one compound. Primary annotation was performed by matching experimental MS/MS spectra against the GNPS spectral database.

FBMN [19] was applied to the samples and features (nodes) were connected by a similarity score of their MS/MS spectra (cosine score ≥ 0.7 with at least 4 matched signals). Default settings given by GNPS for FBMN are cosine ≥ 0.7 and 6 matched signals [8]. The default settings are given for natural compounds of a possible higher mass than anthropogenic substances present in river water. The number of expectable fragments is lower, though, in smaller molecules. Therefore, the number of matched signals to enable connection of features was set to 4. Less than 4 signals would lead to probably more results, but also reduce the certainty of the annotated results. Regarding the cosine, the default settings were chosen since a lower cosine (< 0.7) would again lead to more connections but less certainty. Cosines above 0.7 can be individually judged later as they are given for each connection as the strength of the bond between two nodes.

### Evaluation of networks

The resulting networks were exported to Cytoscape [32] and the depiction of networks was customized. The identified and unidentified nodes were visualized as squares and circles, respectively, with their size being dependent on their summed up feature area. The weight of the edges between the nodes was set according to their cosine similarity score and labeled with the m/z difference between the nodes. Area variations between the sample groups, either based on the attributes “Sample Type” or “Season,” were depicted in pie charts within the nodes.

### Quality assurance

To assure the stability of the chromatography and the mass spectrometry, the abovementioned internal standards were used. Maximum time deviation of the internal standards ranged between 0.7 and 1.1% and signal intensity’s relative standard deviations ranged between 8.4 and 13.1% for the internal standards (ESM Table S2). Mass accuracy was <0.005 m/z or 10 Da. Between the measurement of each triplicate, laboratory blanks were included in the sequence. For the subsequent data evaluation, only 10 representative blank samples were considered. Features appearing in blanks in signal intensities higher than the tripled noise level were excluded from the subsequent feature lists of the samples.

## Results and discussion

### Creation and prioritization of networks

**Fig. 1:**
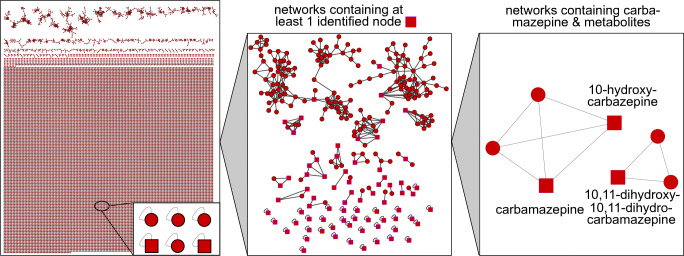
All resulting nodes (red dots) and networks for feature-based molecular networking of river and abstraction well samples (left) with network size decreasing from top to bottom (singletons); abstracted identified nodes and networks containing at least one annotated node (middle); networks containing carbamazepine and related compounds (right)

In total, 19,246 feature nodes containing MS/MS spectra were created for all samples (Fig. 1). Despite filtering ^13^C isotope patterns, these nodes include multiple ion adducts and in-source fragments for one compound and are therefore not equivalent to the number of detected compounds. In total, 507 networks were created containing at least two nodes out of which 375 consisted of exactly two nodes. The largest network contained 86 nodes. A more comprehensive view on the detected features concerning their total number and appearance in different seasons as well as a comparison of the various locations is described in Oberleitner et al., 2020b [33] (for a detailed view, use the URL provided at the end of this document to access the annotated network raw data).

Many nodes remained unconnected from any networks either because of a lack of similar compounds within the samples or insufficient quality of their corresponding MS/MS spectrum. A minimum of four signals is required for a similarity score. Therefore, most nodes form so-called singletons.

Considering only networks that contained nodes with library matches (squares), only 256 nodes remained (1.3% of all feature nodes). Similarly, most annotated nodes were singletons. The largest network contained 53 nodes with 128 connecting edges with annotations for dibutyl phthalate and diethyl phthalate. These are expected to derive from contamination during sampling and measurement from tubes containing phthalate plasticizers. These signals also occurred in blank samples with significantly lower signal intensity. Therefore, there might also be phthalate pollution in the samples derived from the river water. The same can be seen for triphenylphosphine oxide and triphenylphosphate although they did only form singletons and not larger networks. Each network can be rearranged and investigated individually. For further evaluation, two networks with a maximum size of ten nodes were chosen exemplarily. Additionally, networks that contained related annotations (e.g., other sartans) were added to the view of the respective network.

### Carbamazepine and transformation products

**Fig. 2:**
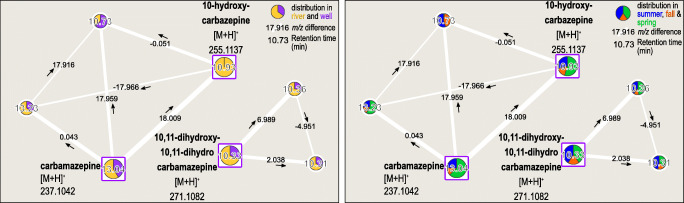
Molecular networks contain carbamazepine and related compounds with distribution in river and abstraction well samples (left) and seasonal distribution (right). Compounds are labeled with their respective retention time. Edge-width amounts to the cosine score. Identified compounds are highlighted as squares and labeled with the respective ion type and *m*/*z* value. Arrows mark the *m*/*z* difference direction

The first identified network is depicted in Fig. 2. Three nodes were annotated as carbamazepine, 10-hydroxycarbazepine, and 10,11-dihydroxy-10,11-dihydro-carbamazepine. Carbamazepine was already determined within the samples in our previous study being mostly persistent during RBF [25]. 10-Hydroxycarbazepine has a high structural similarity to carbamazepine (ESM Fig. S2) and was only found within the river samples; hence, it is well removed during RBF. It is a metabolite of oxcarbazepine previously detected in surface waters but not detected in our study [34]. The seasonal distribution showed a typical distribution for wastewater-derived micropollutants: The highest intensities were detected in summer 2017 and spring 2018. These two sampling times showed the lowest sampled discharge (Fig. 2 in [25]), and therefore the water body consists of a high TWW proportion [30].

10,11-Dihydroxy-10,11-dihydro-carbamazepine is a metabolite of carbamazepine [35] known to be formed during wastewater treatment [36] with distribution within rivers and wells comparable to carbamazepine. Both TP have not yet been investigated concerning their behavior during RBF before. To verify their identification, 10-hydroxycarbazepine and 10,11-dihydro-10,11-dihydroxycarbamazepine were measured as reference standards at three different concentrations (100, 500, and 1000 ng/L). The semi-quantitative analysis regarding the three calibration steps (exact mass, retention time, six to seven signals per peak) showed that river concentrations were below 100 ng/L for 10-hydroxycarbazepine and below 150 ng/L for 10,11-dihydroxy-10,11-dihydro-carbamazepine. Since 10,11-dihydroxy-10,11-dihydro-carbamazepine is only partly removed during RBF, it is relevant to drinking water production in these concentrations and should be observed during the subsequent treatment steps.

### Sartans and their transformation products

**Fig. 3:**
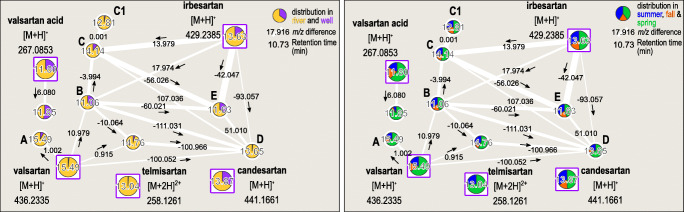
Molecular networks contain sartans and related compounds with distribution in river and abstraction well samples (left) and seasonal distribution (right). Compounds are labeled with their respective retention time. Edge-width amounts to the cosine score. Identified compounds are highlighted as squares and labeled with the respective ion type and *m*/*z* value. Arrows mark the *m*/*z* difference direction. A, valsartan ^13^C isotope; B, olmesartan; C, IRB_442_C; C1, IRB_442_C1; D, dealkylated valsartan; E, dealkylated irbesartan

The second investigated network (Fig. 3) contains nodes annotated as valsartan, telmisartan, candesartan, irbesartan, and their TP valsartan acid by the GNPS database due to their spectral similarity. Annotation was secured by a search for diagnostic fragments and later proven by reference standards. Sartans are a group of antihypertensive drugs with a high production volume (>100 t/a in Germany [37]) and structural similarities (ESM Figure S3), such as biphenyl, imidazole, or tetrazole groups.

Nodes **A**–**E** were not annotated during spectral library search but further investigated in this study and manually annotated as a valsartan ^13^C isotope, olmesartan, an oxygenated form of irbesartan IRB_442 and a structural isomer (C1), dealkylated valsartan, and dealkylated irbesartan, respectively. An identification level 2 was achieved for nodes **A**–**E** as described by the metabolomics standards initiative (MSI) and Schymanski et al. [2]. This means that **A**–**E** are probable structures annotated by diagnostic fragments. For a level 1 confirmed structure, a reference standard would be needed, often unavailable for transformation products. Compounds at 14.76 and 11.85 min were not annotated in this study and remain unidentified. Yet, they are very likely sartan-related compounds that show similar fragment spectra, but are not yet described in literature.

#### Identification of transformation products

Node **A** showed a spectral similarity to valsartan, the same retention time (15.49 min), and a mass difference of 1.002 Da. Therefore, it was identified as a valsartan ^13^C isotope that was missed during isotope filtering in MZmine.

With an accurate *m*/*z* of 447.212 and a retention time of 11.06 min, node **B** fitted the molecular formula C_24_H_27_N_6_O_3_^+^ (4 ppm tolerance). Literature research identified it as olmesartan [38] and a spectral mirror match (ESM Figure S4) confirmed the identification via the spectral library MassBank of North America (MoNA) [39], although the measured mass spectrum had a low intensity and only fragments between *m*/*z* 177 and 235.

Node **C** showed high similarity to irbesartan and a mass difference of +13.979 Da (t_R_ = 14.14 min). Literature research towards TP of irbesartan resulted in an oxygenated form of irbesartan IRB_442 [40]. The annotation was confirmed by a spectral investigation (ESM Figure S5). The fragment at *m*/*z* 235 resulted from a loss of the imidazole structure, followed by a loss of N_2_ to a fragment at *m*/*z* 207. Fragment *m*/*z* 180 is the biphenyl-structure with a bonded CN part. Node **C1** has the same parent mass and fragments as **C** but an earlier retention time (12.81 min). Therefore, we annotated it as a structural isomer of IRB_442_C as found before by Boix et al. [41].

Node **D** was annotated by the similarity to valsartan and the mass difference of −100.052 Da as a dealkylated valsartan which was previously described as a transformation product of valsartan [42]. It showed the same diagnostic fragments as IRB_442_C (ESM Figure S6).

Compound **E** showed a high spectral similarity to irbesartan and IRB_442 and a mass difference of −42.047 Da to irbesartan. Additional to fragments at *m*/*z* 207 and 180, fragments at *m*/*z* 344 and 153 (ESM Figure S7) were diagnostic for a dealkylated TP of irbesartan (-propylene) [41].

The retention behavior of the compounds was compared to different RP-HPLC-MS studies [38, 41–43] and, except for candesartan, the elution order of the sartans was conclusive (ESM Figure S8). Candesartan eluted between irbesartan and valsartan, but in literature, it was either found to elute before [38] or after [43] both compounds depending on the applied column, as mobile phases were the same in both studies (water and methanol with 0.1% FA). Therefore, we assumed that candesartan elution was very sensitive to the chosen column and chromatographic conditions, e.g., the chosen eluents.

The identification via diagnostic fragments and literature data leads to an identification level of two (probable structure) for the compounds **A**–**E** [2]. Retention behavior was conclusive and the presence of the sartans makes the occurrence of their TP probable [2]. The identification of valsartan, valsartan acid, irbesartan, candesartan, olmesartan, and telmisartan was verified by reference standards to level 1. The semi-quantitative analysis showed concentrations for all sample types of <100 ng/L for irbesartan and telmisartan, mean concentrations <100 ng/L (maximum 200–300 ng/L) for candesartan and valsartan, mean concentrations of 400 ng/L (maximum ~425 ng/L) for olmesartan, and mean concentrations of 200–300 ng/L (maximum 600–700 ng/L) for valsartan acid. For valsartan, concentrations between 0.5 and 150 ng/L have been reported in British rivers before [44]. For Bavarian river catchments, mean concentrations in WWTP effluents of 460 ng/L for candesartan, 1250 ng/L for irbesartan, 740 ng/L for olmesartan, 680 ng/L for telmisartan, and 1100 ng/L for valsartan are known [38]. Considering the dilution of TWW in the Ems river and Ruhr river at different discharge scenarios and a different proportion of prescribed sartans per region, this results in comparable river water concentrations as mentioned for WWTP effluents. In the Ems river, telmisartan and irbesartan were frequently detected in a round robin test, as well as olmesartan, telmisartan, and irbesartan in the Ruhr river, which is in line with our frequent detection of the compounds [45]. Nödler et al. (2013) found a maximum concentration of 2119 ng/L (Creek Gonna) for valsartan acid (median 65 ng/L of 13 surface water samples across Germany) in surface water [46], which is a larger range than our results (<LOD - 700 ng/L) indicating a medium wastewater impact for the Ruhr and Ems rivers compared to other German rivers. This is in line with Karakurt et al., calculating a TWW proportion of around 50–100% at low river discharge and far less than 50% at medium discharge [30]. Valsartan acid was also suggested as a possible wastewater indicator alongside carbamazepine as it showed persistence and a correlation to carbamazepine concentration in surface water. It is concluded that the identified sartans at these concentration levels are of particular interest concerning drinking water production from riverbank filtrate. They should be included in routine analysis more frequently.

#### Seasonal occurrence and elimination during riverbank filtration under different redox conditions

Valsartan, telmisartan, and compounds **A**, **C1**, and **D** and the unspecified compound at 14.76 min were almost exclusively found in the river samples and are therefore not relevant for drinking water production. Valsartan acid, irbesartan, candesartan, and compounds **B**, **C**, and **E** and an unspecified compound at 11.85 min were also found in the well samples and showed a typical seasonal distribution of wastewater-derived OMP [47].

**Fig. 4:**
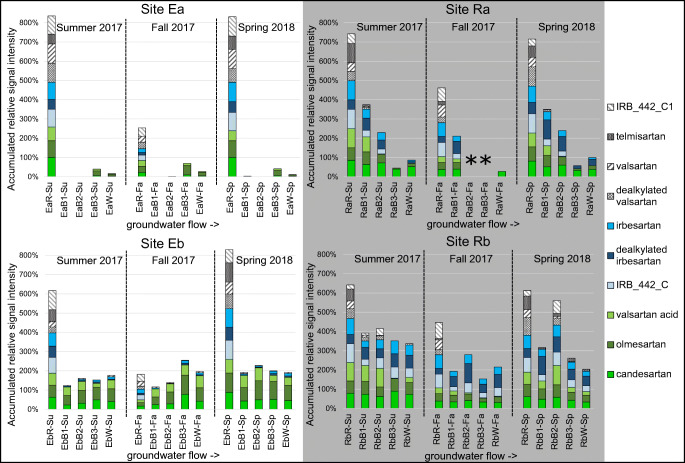
Seasonal distribution of sartans and related compounds in riverbank filtration systems at Ems river (left) and Ruhr river (right) as accumulated relative signal intensities. The highest peak area of each compound was set to 100%. Ea, Ems A; Eb, Ems B; Ra, Ruhr A; Rb, Ruhr B; R, river; B1-B3, groundwater wells; W, abstraction well; Su, summer; Fa, fall; Sp, spring. The asterisk symbol indicates sampling in fall 2017 not possible due to low groundwater table

Intensities for each of the sartans were normalized to the corresponding maximum intensity across all samples from the different locations, which was set to 100%. Matrix effects were not investigated in this study but in regard to our previous study (Oberleitner et al., 2020 [25]), major matrix effects influencing the distribution of the analytes were not expected. The resulting distribution of the sartans and their TP is depicted in Fig. 4.

Compounds IRB_442_C1, telmisartan, valsartan, and dealkylated valsartan are depicted as gray-hatched bars. Maximum intensities for these compounds were found in the Ems river at site Ea. At both Ems river sites, intensities decreased in fall due to dilution by precipitation (river discharge ratio summer:fall ~ 1:2) [48]. At the Ruhr river, only telmisartan and dealkylated valsartan show decreased intensities in fall, whereas IRB_442_C1 and valsartan show increased abundances in fall. A possible explanation could be increased photodegradation in spring and summer [46, 47]. However, possible photodegradation was also reported for telmisartan [49], candesartan [50], and irbesartan [51] which show a different seasonal distribution. The four compounds were found in all river samples but were immediately eliminated after entering the hyporheic zone and therefore not or only in small intensities found in the first groundwater wells near the bank (B1). In our study, the elimination of these compounds was not dependent on redox conditions which was reported for valsartan [52]. Valsartan was also previously found to be persistent during RBF to some extent, which is conclusive with our findings [52, 53].

Irbesartan, dealkylated irbesartan, and IRB_442_C are depicted in shades of blue and were found in all river samples in comparable intensities, except for fall river samples of the Ems river. Here, the before-mentioned dilution is expected to be responsible. Notably, these compounds were found in the well samples (B1-W) of the Ruhr river sites in comparable or slightly decreased intensities in contrast to their presence only in small intensities in the wells of the Ems river sites. Since the Ems river sites are both anoxic, it is assumed that the elimination of irbesartan and its TP is increased under anoxic conditions. In contrast, irbesartan was previously found to be persistent to some extent independent from the prevailing redox conditions [52]. TP IRB_442_C is not found in the remote wells (B3 and W) of site Ra, which possibly derives from the longer travel distance (66 m) compared to site Rb (38 m).

Valsartan acid, olmesartan, and candesartan are shaded in green and found in all river samples. Decreased intensities are again found in fall. During RBF, olmesartan and candesartan are partially removed at sites Ea and Ra but not significantly at sites Eb and Rb. This coincides with the respective longer travel distance for both redox conditions. Both have previously been described to be persistent during RBF [54, 55]. Although valsartan acid shows varying intensities in the wells at site Rb, intensities tend to decrease during RBF at the oxic Ruhr river sites and the more anoxic site Ea. Contrary to literature data [52, 53] where valsartan acid was found to be persistent independent from redox conditions, in our study, valsartan acid is partially removed under rather oxic conditions.

To the best of our knowledge, this is the first report on RBF behavior for the TP IRB_442_C and C1, dealkylated valsartan, and dealkylated irbesartan.

### Further identified compounds

As a result of the application of FBMN on surface and groundwater samples from two river systems, a list of 43 components (ESM Table S3, including the before-mentioned micropollutants) was annotated by matches with the GNPS spectral libraries of which five were known targets (metoprolol, diclofenac, oxazepam, caffeine, and carbamazepine) from our previous study [25]. Components that were only present in the river samples, but not in the abstraction wells, are ß-carboline-1-propionic acid (probably derived from plants [56]), 10-hydroxycarbazepine, phenylalanine-leucin (dipeptide), and flufenacet (herbicide). These components are therefore well removed during RBF at all sites. Flufenacet was reported to be eliminated during RBF up to 60% [57]. Flufenacet was only found at the Ems river as well as flurtamone (herbicide) and 7-chloro-3-methylquinoline-8-carboxylic acid (quinmerac, herbicide), which were also found in the abstraction wells of the Ems river sites. These micropollutants are therefore site-specific for the Ems river. Further pharmaceuticals (e.g., sitagliptin, amisulpride, and clindamycin), biocides (e.g., terbutryn), and metabolites (e.g., 2-hydroxyibuprofen and desethylterbutylazine) were common components for all sites and seasons. Natural organic compounds (e.g., l-tryptophane and oleoylserotonin) were found among the identified features. Some of these compounds were previously found to be persistent during RBF to some extent (compare [58–61]). Diclofenac and tris(3-chloropropyl) phosphate were identified as the ^37^Cl isotope of their monoisotopic masses, emphasizing the importance of collecting MS/MS spectra of molecules containing two or more chlorine atoms. Due to the settings in MZmine, the most abundant isotope was selected for the feature list. In molecules containing two or more chlorine atoms, this mass is different from the monoisotopic mass.

## Conclusions

FBMN is a powerful tool to promote the identification of unknowns in environmental water samples at low concentrations. With spectral database search, a total of 43 compounds was annotated. Additionally, in comparison to other identification tools, FBMN enables the annotation of unknown species with spectral similarity search based on known annotated compounds present in the sample. Therefore, it broadens the annotation of unknowns in non-target analysis towards (unknown) transformation products at low concentrations that cannot be identified by database search due to lacking spectral data. In this case, several transformation products of sartans were annotated by their spectral similarity to their parent compounds although a spectral database search did not lead to any hits. Still, it has to be considered that thousands of features present in the samples could not yet be annotated due to lack of intensity or fragment spectra and the fact that the GNPS database is still focused on natural compounds. The number of annotated compounds could eventually increase by extending the database towards anthropogenic pollutants.

Another advantage over other molecular networking tools is the utilization of chromatographic data enabling a plausibility check of annotated features to their retention time which in this case confirmed the identification of sartan TP.

## Supplementary Information

ESM 1 (DOCX 1248 kb)

## Data Availability

The annotated network raw data can be accessed through https://gnps.ucsd.edu/ProteoSAFe/status.jsp?task=a35c2a4e0408402389c48d78ab692b7a. Please note that the raw data has later been cleared of double hits and obvious false positives in the subsequent Cytoscape visualization.
