# Supplementary material for: Feature-based molecular networking for identification of organic micropollutants including metabolites by non-target analysis applied to riverbank filtration
Source: Anal Bioanal Chem. 2021 Jul 20;413(21):5291–300. doi: 10.1007/s00216-021-03500-7 (PMC8405475; doi:10.1007/s00216-021-03500-7)
Supplement: Supplementary file 1 — (DOCX 1248 kb) [file 216_2021_3500_MOESM1_ESM.docx]

**Supplementary Information**

**Feature-based molecular networking for identification of organic micropollutants including metabolites by non-target analysis applied to riverbank filtration**

**Analytical and Bioanalytical Chemistry**

*Daniela Oberleitner^a^, Robin Schmid^b^, Wolfgang Schulz^c^, Axel Bergmann^d^, Christine Achten^a,*^*

^a^Institute of Geology and Palaeontology – Applied Geology, University of Münster, Corrensstraße 24, 48149 Münster, Germany

^b^Institute of Inorganic and Analytical Chemistry, University of Münster, Corrensstraße 28/30, 48149 Münster, Germany

^c^Laboratory for Operation Control and Research, Zweckverband Landeswasserversorgung, Am Spitzigen Berg 1, 89129 Langenau, Germany

^d^Rheinisch-Westfälische Wasserwerksgesellschaft mbH, Am Schloß Broich 1-3, 45479 Mülheim (Ruhr), Germany

*Corresponding author. E-mail address: achten@uni-muenster.de

**Supplementary Information:** Tables of the MZmine 2.51 settings for non-target analysis and list of annotated features; Figures of geological cross-sections, chemical structures of carbamazepine, oxcarbazepine and transformation products, chemical structure of selected sartans, mirror plot of olmesartan and compound B, diagnostic fragments of compounds C - E, chromatogram of sartans and compounds A - E.

**Table S1.** Parameters for data processing with MZmine 2.51 for positive ionization mode data from a Bruker Maxis 3G qTOF.

| Parameter | Value |
| --- | --- |
| Crop Filter | 5 - 22 min |
| Mass detection |  |
| MS Level | 1 |
| Mass detector | Centroid |
| Noise Level | 300 |
| Mass detection |  |
| MS Level | 2 |
| Mass detector | Centroid |
| Noise Level | 200 |
| ADAP Chromatogram builder |  |
| MS Level | 1 |
| Min. group size in # of scans | 3 |
| Group intensity threshold | 300 |
| Min highest intensity | 800 |
| m/z tolerance | 0.002 *m/z* or 10 ppm |
| Smoothing |  |
| Filter width | 5 |
| Chromatogram deconvolution |  |
| Local minimum search |  |
| Chromatographic threshold | 90% |
| search minimum RT range | 0.08 min |
| minimum rel. Height | 0% |
| minimum absol. Height | 800 |
| ratio top/edge | 1.3 |
| peak duration | 0.03 - 2 min |
| m/z center calculation | MEDIAN |
| m/z range for MS2 scan pairing (Da) | 0.1 |
| RT range for MS2 scan pairing (min) | 0.1 |
| Join aligner |  |
| m/z tolerance | 0.003 *m/z* or 10 ppm |
| weight for m/z | 3 |
| retention time tolerance | 0.15 min |
| weight for retention time | 1 |
| Peak list rows filter |  |
| Keep only peaks with MS2 scan (GNPS) | yes |
| Peak finder (multithreaded) |  |
| Intensity tolerance | 0% |
| m/z tolerance | 0.0015 *m/z* or 10 ppm |
| RT tolerance | 0.1 min |
| Filter |  |
| Duplicate  Height  Data points  Duration |  |
| Export for/Submit to GNPS |  |
| Merge MS/MS (experimental) | yes |
| Select spectra to merge | Across samples |
| m/z merge mode | Weighted average (remove outliers) |
| Intensity merge mode | Sum intensities |
| Expected mass deviation | 0.002 *m/z* or 15 ppm |
| Cosine threshold (%) | 70 |
| Peak count threshold (%) | 20 |
| Isolation window offset (m/z) | 0 |
| Isolation window width (m/z) | 3 |
| Filter rows | Only with MS2 |
| Presets | Highres |

**Table S2.** Internal Standards and respective parameters used for quality assurance of the utilized method.

| Substance | *m/z* | Max. time deviation | Relative standard deviation |
| --- | --- | --- | --- |
| Sulfadimethoxine d6 | 317.1186 | 0.8% | 13.1% |
| Diuron d6 | 239.0620 | 0.7% | 8.4% |
| Benzotriazole d4 | 124.0808 | 1.1% | 12.7% |

**Table S3.** List of annotated features. Score ≥ 0.7, *m/z* error ≤ 10 ppm.

| No. | Ion | Name | Compound class | *m/z* error (ppm) | Score | Location | River | Sample Type | Season | *m/z* | RT (min) |
| --- | --- | --- | --- | --- | --- | --- | --- | --- | --- | --- | --- |
| Only in river samples | | | | | | | | | | | |
| 1 | [M+H]+ | ß-Carboline-1-propionic acid | natural origin | 8.73 | 0.829 | Ra,Rb,Ea,Eb | Ems,Ruhr | River | Spring,Summer,Fall | 241.099 | 9.16 |
| 2 | [M+H]+ | 10-Hydroxycarbazepine | pharmaceutical TP | 3.59 | 0.781 | Ra,Rb,Ea,Eb | Ems,Ruhr | River | Spring,Summer,Fall | 255.114 | 10.93 |
| 3 | [M+H]+ | Phenylalanin-Leucin | natural origin | 9.40 | 0.742 | Rb,Ea,Eb | Ems,Ruhr | River | Spring,Summer,Fall | 279.167 | 9.35 |
| 4 | [M+H]+ | Flufenacet | herbicide | 3.02 | 0.851 | Ea,Eb | Ems | River | Fall | 364.073 | 17.60 |
|  | | | | | | | | | | | |
| Only at Ems river sites | | | | | | | | | | | |
| 5 | [M+H]+ | Flurtamone | herbicide | 0.82 | 0.930 | Ea,Eb | Ems | River,Well | Spring,Summer,Fall | 334.105 | 16.04 |
| 6 | [M+H]+ | 7-Chloro-3-methylquinoline-8-carboxylic acid (quinmerac) | herbicide | 0.48 | 0.826 | Ea,Eb | Ems | River,Well | Spring,Summer,Fall | 222.032 | 10.87 |
|  | | | | | | | | | | | |
| Common | | | | | | | | | | | |
| 7 | [M+H]+ | Carbamazepine | pharmaceutical | 0.84 | 0.944 | Ra,Rb,Ea,Eb | Ems,Ruhr | River,Well | Spring,Summer,Fall | 237.104 | 13.04 |
| 8 | [M(^37^Cl) +H]+ | Tris(3-chloropropyl) phosphate | industrial chemical | 8.53 | 0.944 | Ra,Rb,Ea,Eb | Ems,Ruhr | River,Well | Spring,Summer,Fall | 329.003 | 16.16 |
| 9 | [M+H]+ | Sitagliptin | pharmaceutical | 1.50 | 0.936 | Ra,Rb,Ea,Eb | Ems,Ruhr | River,Well | Spring,Summer,Fall | 408.126 | 10.14 |
| 10 | [M+H]+ | Diethyl phthalate | plasticizer | 3.56 | 0.912 | Ra,Rb,Ea,Eb | Ems,Ruhr | River,Well | Spring,Summer,Fall | 223.096 | 19.81 |
| 11 | [M+H]+ | Candesartan | pharmaceutical | 2.01 | 0.911 | Ra,Rb,Ea,Eb | Ems,Ruhr | River,Well | Spring,Summer,Fall | 441.166 | 13.87 |
| 12 | [M+H]+ | Irbesartan | pharmaceutical | 3.48 | 0.910 | Ra,Rb,Ea,Eb | Ems,Ruhr | River,Well | Spring,Summer,Fall | 429.239 | 13.63 |
| 13 | [M+H]+ | Tiapridal | pharmaceutical | 2.50 | 0.896 | Ra,Rb,Ea,Eb | Ems,Ruhr | River,Well | Spring,Summer,Fall | 329.152 | 7.98 |
| 14 | [M+H]+ | Dibutyl phthalate | plasticizer | 4.70 | 0.894 | Ra,Rb,Ea,Eb | Ems,Ruhr | River,Well | Spring,Summer,Fall | 279.159 | 7.80 |
| 15 | [M+H]+ | Valsartan acid | pharmaceutical TP | 2.63 | 0.887 | Ra,Rb,Ea,Eb | Ems,Ruhr | River,Well | Spring,Summer,Fall | 267.087 | 11.77 |
| 16 | [M+H]+ | Amisulpride | pharmaceutical | 1.90 | 0.881 | Ra,Rb,Ea,Eb | Ems,Ruhr | River,Well | Spring,Summer,Fall | 370.179 | 8.87 |
| 17 | [M+H]+ | Triphenylphosphine oxide | industrial chemical | 1.42 | 0.880 | Ra,Rb,Ea,Eb | Ems,Ruhr | River,Well | Spring,Summer,Fall | 279.093 | 14.66 |
| 18 | [M+H]+ | Oxazepam | pharmaceutical | 8.29 | 0.873 | Ra,Rb,Ea,Eb | Ems,Ruhr | River,Well | Spring,Summer,Fall | 287.056 | 13.40 |
| 19 | [M]+ | Lauramidopropyl betaine (Cocamidopropyl betaines) | surfactant | 3.20 | 0.863 | Ra,Rb,Ea,Eb | Ems,Ruhr | River,Well | Spring,Summer,Fall | 343.295 | 14.30 |
| 20 | [M+H]+ | Desethylterbutylazine | herbicide TP | 1.51 | 0.860 | Ra,Rb,Ea,Eb | Ems,Ruhr | River,Well | Spring,Summer,Fall | 202.085 | 13.09 |
| 21 | [M+H]+ | Phenylbenzimidazole sulfonic acid | sunscreen agent | 1.78 | 0.853 | Ra,Rb,Ea,Eb | Ems,Ruhr | River,Well | Spring,Summer,Fall | 275.049 | 8.26 |
| 22 | [M+H]+ | Valsartan | pharmaceutical | 1.89 | 0.849 | Ra,Rb,Ea,Eb | Ems,Ruhr | River,Well | Spring,Summer,Fall | 436.234 | 15.49 |
| 23 | [M(^37^Cl) +H]+ | Diclofenac | pharmaceutical | 2.36 | 0.843 | Ra,Rb,Ea,Eb | Ems,Ruhr | River,Well | Spring,Summer,Fall | 298.021 | 16.74 |
| 24 | [M+H]+ | Oleoylserotonin | natural origin | 5.46 | 0.824 | Rb,Ea,Eb | Ems,Ruhr | River,Well | Spring,Summer,Fall | 441.350 | 21.97 |
| 25 | [M+H-NH3]+ | 2,7-Diaminofluorene | industrial chemical | 2.20 | 0.820 | Ra,Rb,Ea,Eb | Ems,Ruhr | River,Well | Spring,Summer,Fall | 180.080 | 9.30 |
| 26 | [M+H]+ | Clindamycin | pharmaceutical | 7.54 | 0.809 | Ra,Rb,Ea,Eb | Ems,Ruhr | River,Well | Spring,Summer,Fall | 425.184 | 10.35 |
| 27 | [M]+ | Tetrabutylammonium cation | industrial chemical | 1.64 | 0.809 | Ra,Rb,Ea,Eb | Ems,Ruhr | River,Well | Spring,Summer,Fall | 242.284 | 13.18 |
| 28 | [M+H]+ | Torsemide | pharmaceutical | 6.29 | 0.805 | Ra,Rb,Ea,Eb | Ems,Ruhr | River,Well | Spring,Summer,Fall | 349.131 | 11.42 |
| 29 | [M+H]+ | L-Tryptophan | natural origin | 1.93 | 0.794 | Ra,Rb,Ea,Eb | Ems,Ruhr | River,Well | Spring,Summer,Fall | 205.097 | 7.78 |
| 30 | [M+H]+ | Triphenylphosphate | plasticizer | 0.93 | 0.791 | Ra,Rb,Ea,Eb | Ems,Ruhr | River,Well | Spring,Summer,Fall | 327.078 | 18.49 |
| 31 | [M+H]+ | Levocetirizine | pharmaceutical | 1.80 | 0.789 | Ra,Rb,Ea,Eb | Ems,Ruhr | River,Well | Spring,Summer,Fall | 389.161 | 12.73 |
| 32 | [M+H]+ | 2'-deoxy-guanosine | natural origin | 1.48 | 0.787 | Ra,Rb,Ea,Eb | Ems,Ruhr | River,Well | Spring,Summer,Fall | 268.104 | 6.69 |
| 33 | [M+H]+ | 10,11-trans-Dihydroxy-10,11-dihydrocarbamazepine | pharmaceutical TP | 0.68 | 0.785 | Ra,Rb,Ea,Eb | Ems,Ruhr | River,Well | Spring,Summer,Fall | 271.108 | 10.33 |
| 34 | [M]+ | Trospium | pharmaceutical | 3.03 | 0.784 | Ra,Rb,Ea,Eb | Ems,Ruhr | River,Well | Spring,Summer,Fall | 392.221 | 11.36 |
| 35 | [M+H]+ | Metoprolol | pharmaceutical | 3.07 | 0.762 | Ra,Rb,Ea,Eb | Ems,Ruhr | River,Well | Spring,Summer,Fall | 268.190 | 9.59 |
| 36 | [M+H]+ | Terbutryne | herbicide | 3.72 | 0.756 | Ra,Rb,Ea,Eb | Ems,Ruhr | River,Well | Spring,Summer,Fall | 242.144 | 14.50 |
| 37 | [M+H]+ | O-Desmethylvenlafaxine | pharmaceutical | 1.16 | 0.741 | Ra,Rb,Ea,Eb | Ems,Ruhr | River,Well | Spring,Summer,Fall | 264.195 | 9.12 |
| 38 | [M+H]+ | Hexamethylcyclotrisiloxane | industrial chemical | 1.37 | 0.736 | Ra,Rb,Ea,Eb | Ems,Ruhr | River,Well | Spring,Summer,Fall | 223.063 | 17.80 |
| 39 | [M+2H]2+ | Telmisartan | pharmaceutical | 1.54 | 0.730 | Ra,Rb,Ea,Eb | Ems,Ruhr | River,Well | Spring,Summer,Fall | 258.126 | 13.04 |
| 40 | [M+H-C9H16]+ | all trans-Retinal | natural origin | 0.66 | 0.729 | Ra,Rb,Ea,Eb | Ems,Ruhr | River,Well | Spring,Summer,Fall | 161.096 | 15.68 |
| 41 | [M+H]+ | 5'-Methylthioadenosine | natural origin | 3.99 | 0.722 | Ra,Rb,Ea,Eb | Ems,Ruhr | River,Well | Spring,Summer,Fall | 298.096 | 7.86 |
| 42 | [M+H]+ | Caffeine | pharmaceutical | 5.63 | 0.721 | Ra,Rb,Ea,Eb | Ems,Ruhr | River,Well | Spring,Summer,Fall | 195.087 | 8.68 |
| 43 | [M+H-H2O]+ | 2-Hydroxyibuprofen | pharmaceutical TP | 1.49 | 0.705 | Ra,Rb,Ea,Eb | Ems,Ruhr | River,Well | Spring,Summer,Fall | 205.122 | 12.21 |

**
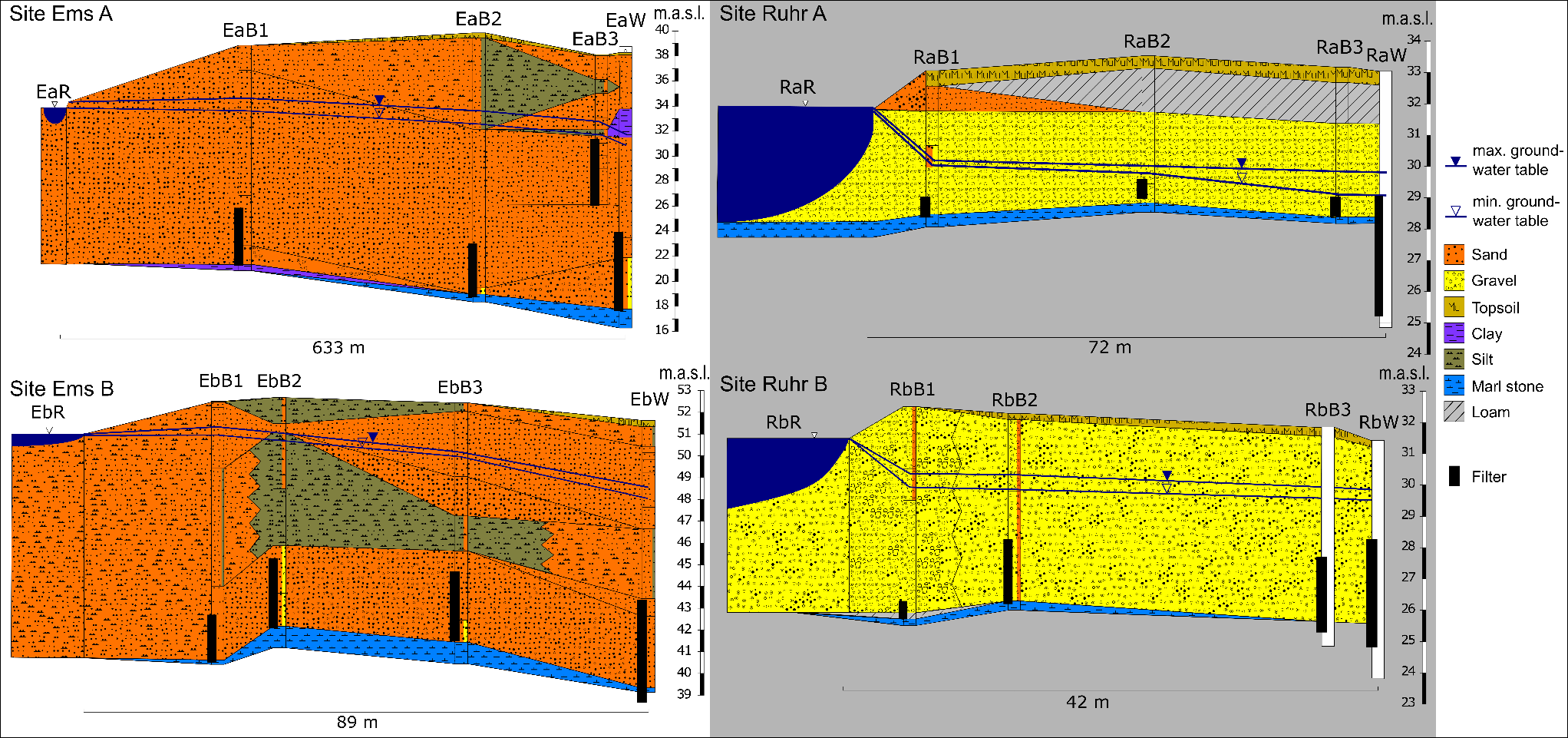
**

**Figure S1.** Geological cross sections of the four investigated locations at Ems river and Ruhr river.

**Figure S2.** Chemical structures of carbamazepine, oxcarbazepine and their transformation products.

**Figure S3.** Chemical structures of a selection of sartans.

**
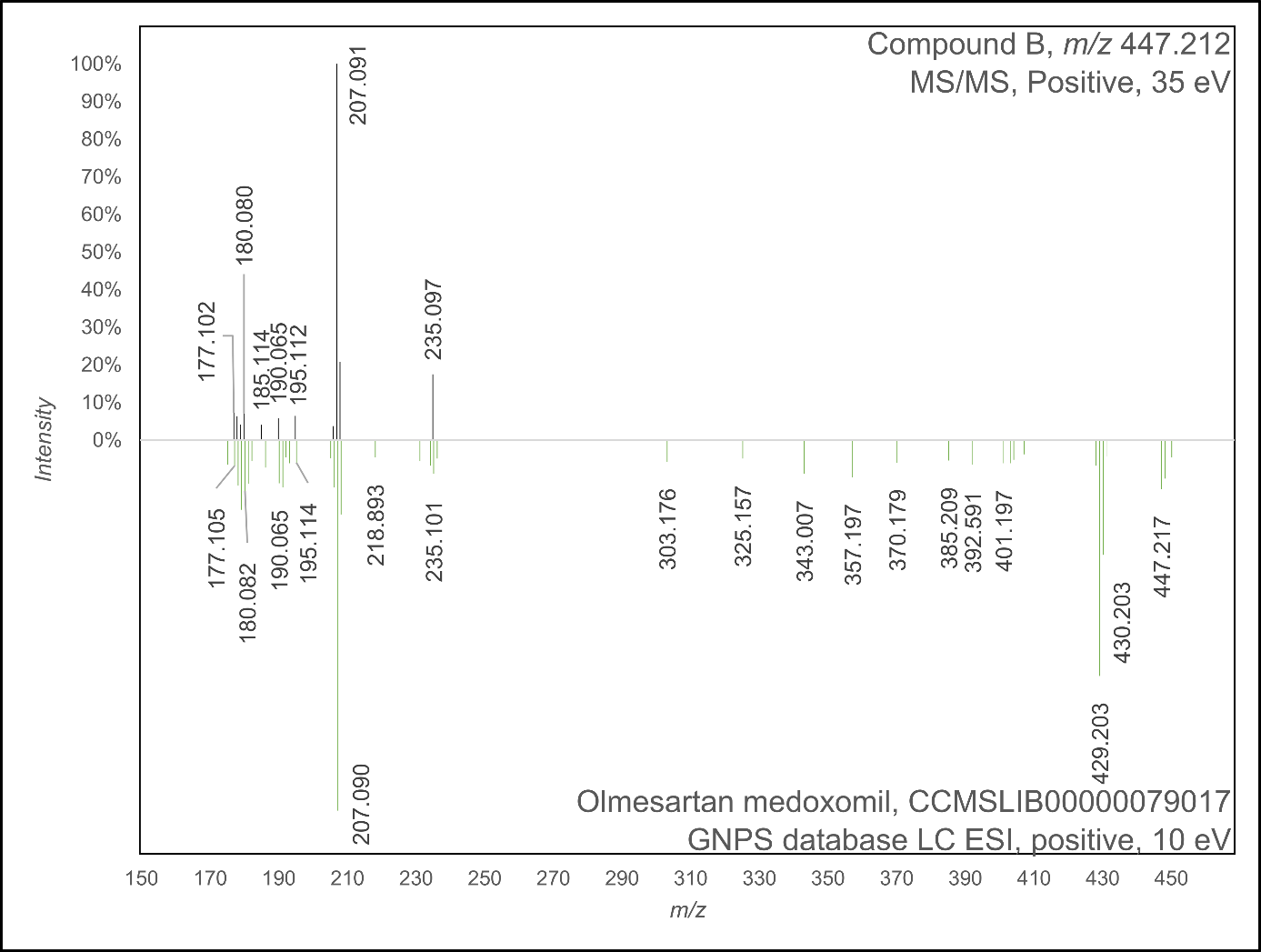
**

**Figure S4.** Database mirror plot of an MS/MS spectrum of compound B and a reference MS/MS spectrum of olmesartan.


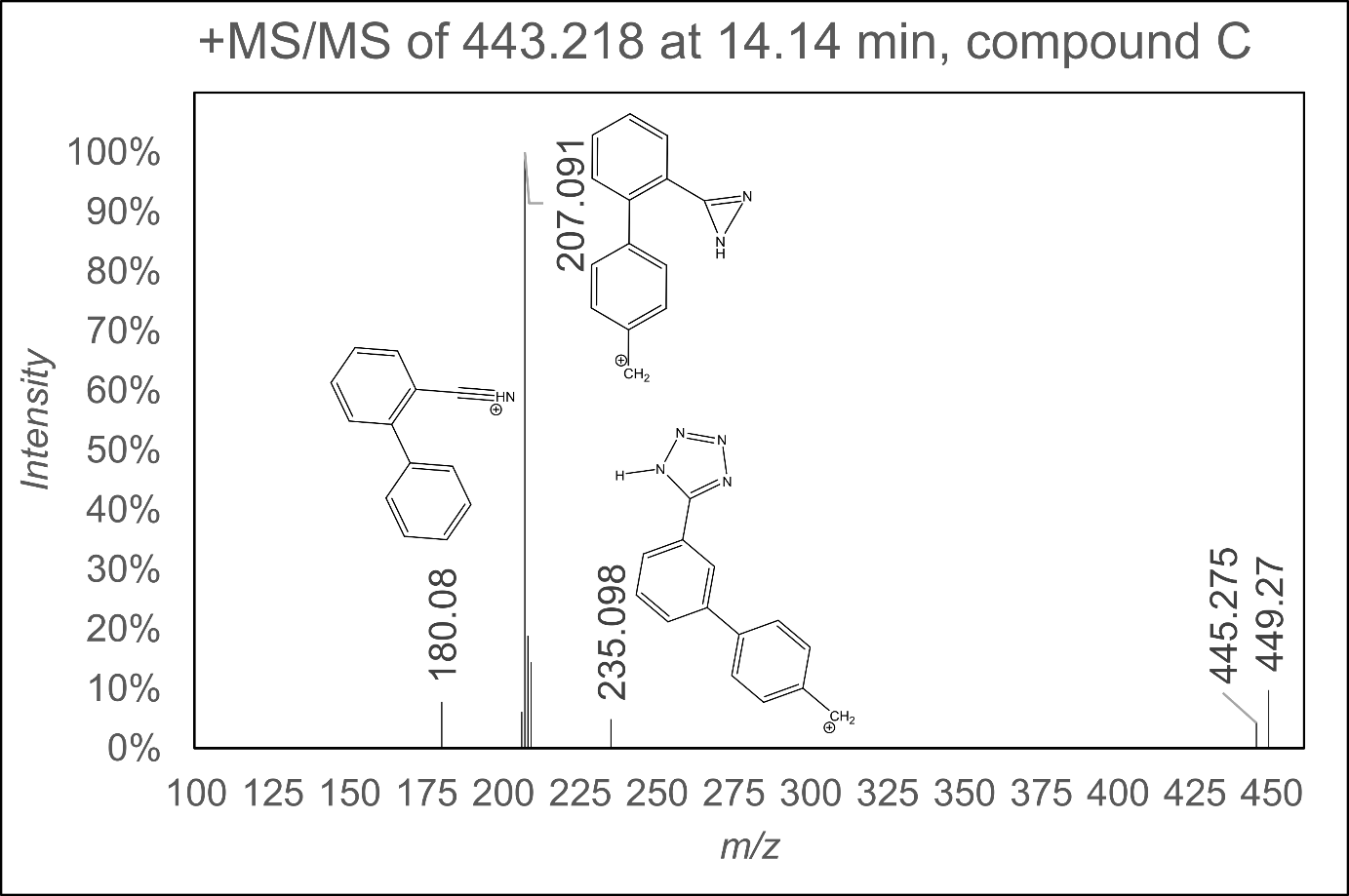


**Figure S5.** Diagnostic fragments of IRB_442_C (compound C).

**
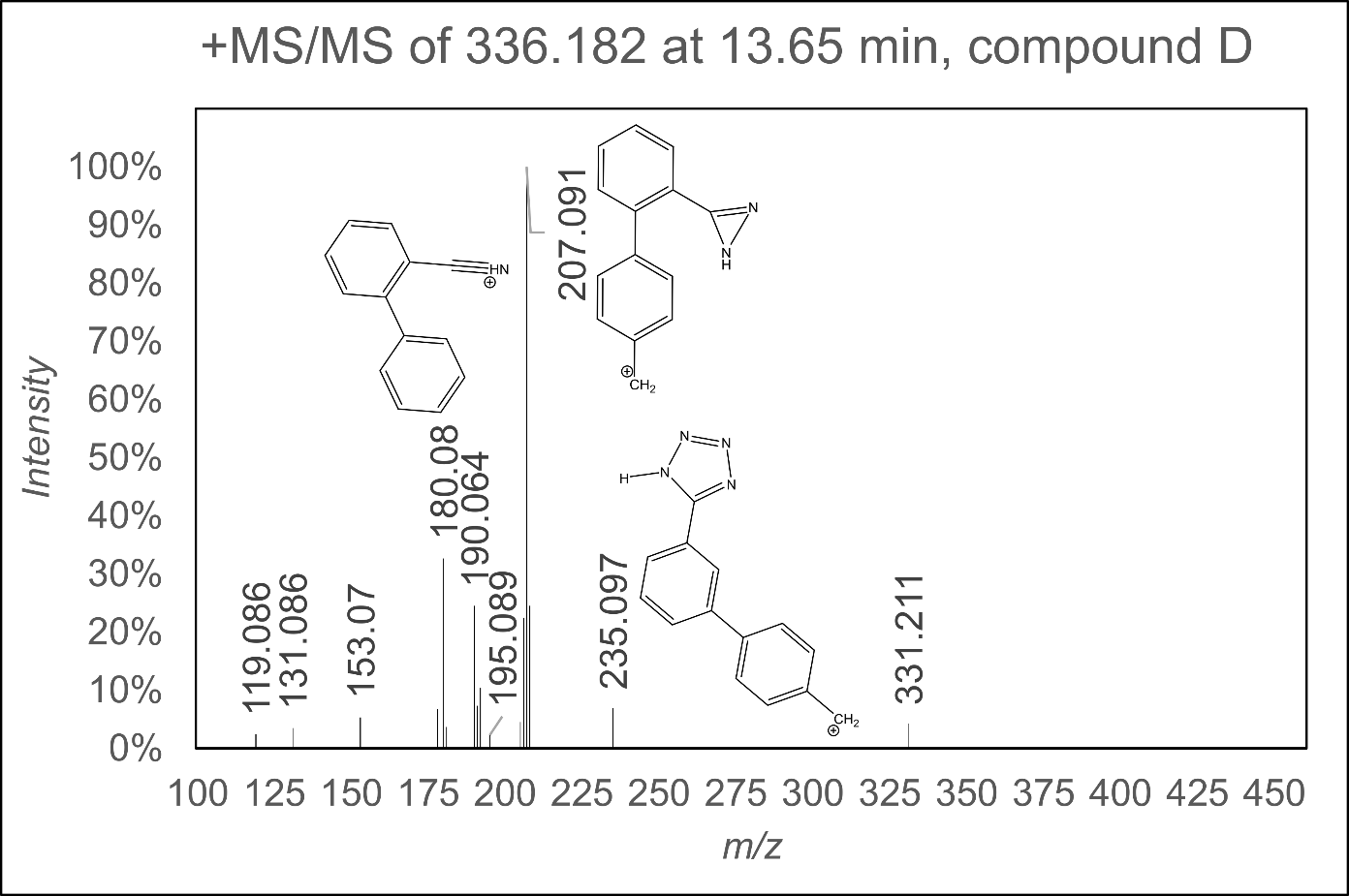
**

**Figure S6.** Diagnostic fragments of dealkylated valsartan (compound D)


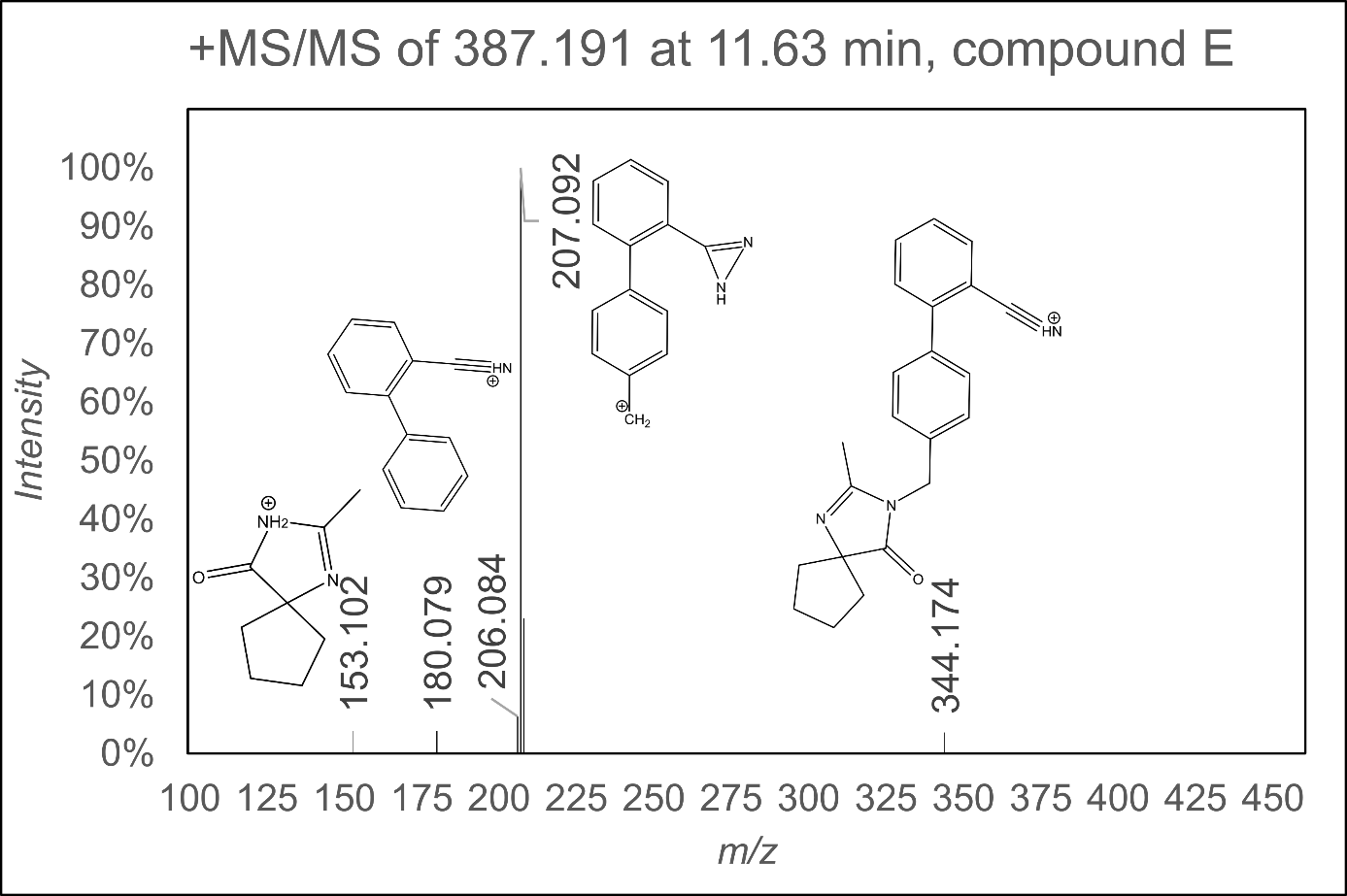


**Figure S7.** Diagnostic fragments of a dealkylated TP of irbesartan (compound E)


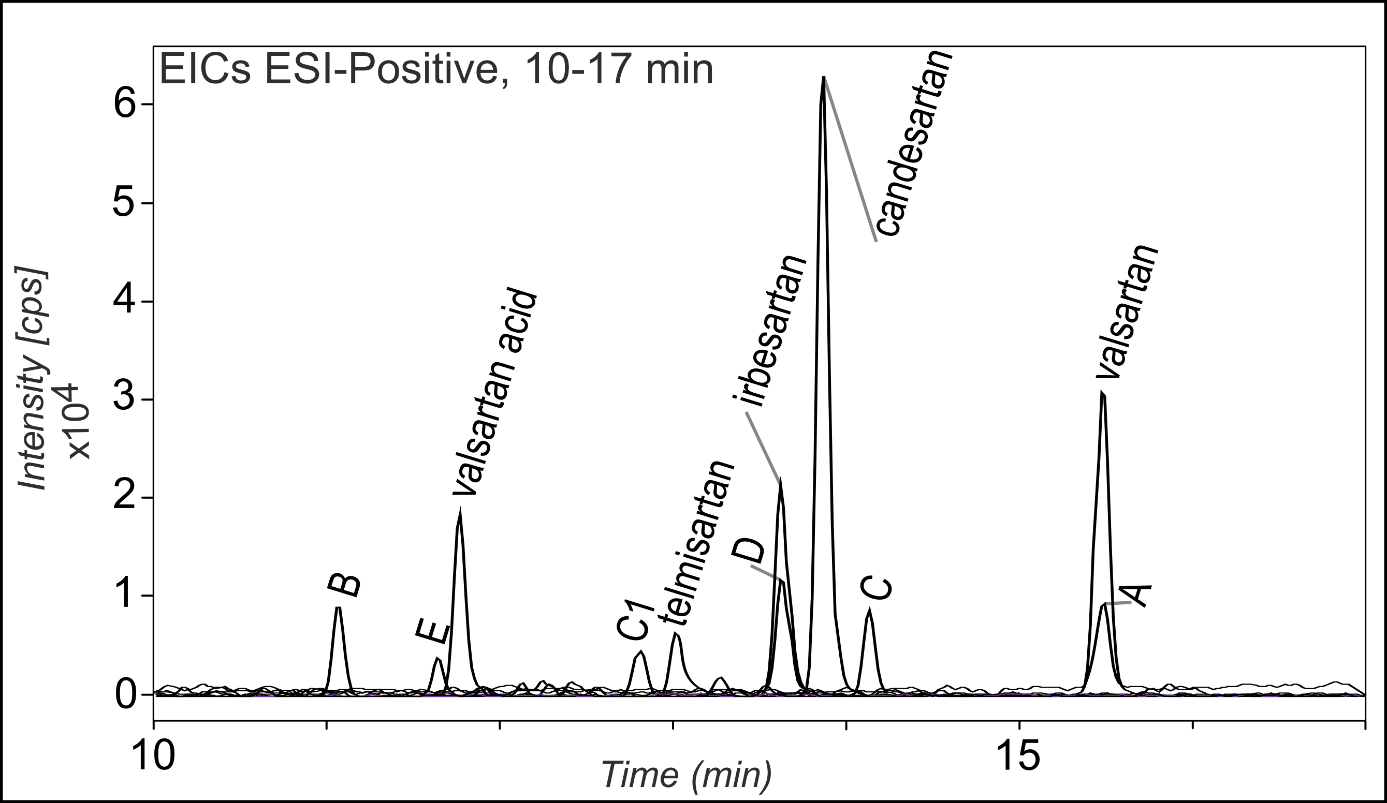


**Figure S8.** Extracted Ion Chromatograms (EIC, 10 - 17 min) of identified sartans and compounds A - E.
